# An overview to process design, simulation and sustainability evaluation of biodiesel production

**DOI:** 10.1186/s13068-021-01977-z

**Published:** 2021-06-01

**Authors:** Mustafa Kamal Pasha, Lingmei Dai, Dehua Liu, Miao Guo, Wei Du

**Affiliations:** 1 Department of Chemical Engineering, Key Laboratory for Industrial Biocatalysis, Ministry of Education, Tsinghua University, Beijing, 100084 China; 2 Department of Engineering, Faculty of Natural and Mathematical Sciences, King’s College London, London, UK; 3 Tsinghua Innovation Center in Dongguan, Guangdong, 523808 China

**Keywords:** Biodiesel, Life cycle analysis, Lipase, Process simulation, Sustainability, Optimization

## Abstract

The overwhelming concerns due to over exploitation of fossil resources necessitate the utilization of alternative energy resources. Biodiesel has been considered as one of the most adaptable alternative to fossil-derived diesel with similar properties and numerous environmental benefits. Although there are various approaches for biodiesel production, development of cost-effective and robust catalyst with efficient production methods and utilization of a variety of feedstock could be the optimum solution to bring down the production cost. Considering the complexity of biodiesel production processes, process design, quantitative evaluation and optimization of the biodiesel from whole systems perspectives is essential for unlocking the complexity and enhancing the system performances. Process systems engineering offers an efficient approach to design and optimize biodiesel manufacturing systems by using a variety of tools. This review reflects state-of-the-art biodiesel research in the field of process systems engineering with a particular focus on biodiesel production including process design and simulation, sustainability evaluation, optimization and supply chain management. This review also highlights the challenges and opportunities for the development of potentially sustainable and eco-friendly enzymatic biodiesel technology.
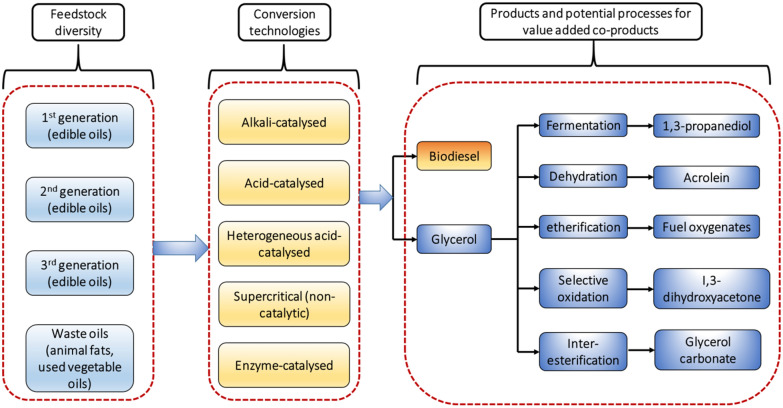

## Introduction

**Fig. 1:**
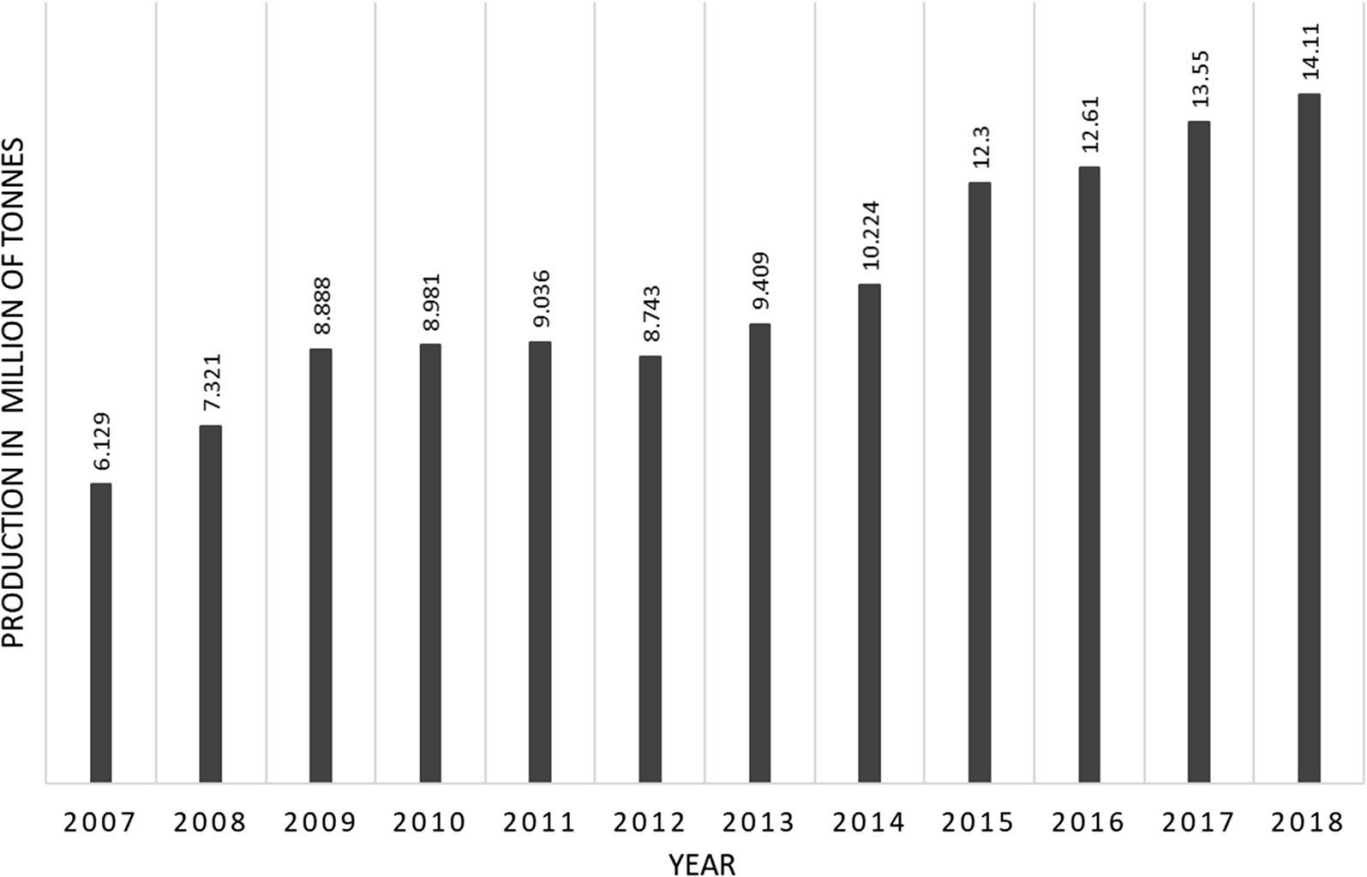
Yearly increase in biodiesel manufacturing in European Union (EU) from 2007 to 2018

Global climate change is threatening the ecosystem worldwide by temperature increase and climate swings. Report published by the Intergovernmental Panel on Climate Change (IPCC) concluded that there is higher probability of about one million species’ extinction if the average global temperature escalates the minimal margin of 1.5 °C [1, 2]. Greenhouse gas (GHG) emissions from anthropogenic activities such as burning of fossil fuel to meet the energy requirement are the major contributor to the temperature rise. It is signposted that by 2050, minimum 40% reduction in GHG emissions is obligatory to sustain the average increase < 1.5 °C [1]. This phenomenon continuously compels the community to search for green alternatives both in energy resources and platform chemicals [3]. One of the primary substitutes to conventional fuels is biodiesel, which received ample attention [4]. American Society for Testing and Materials (ASTM) defined biodiesel as “mono-alkyl esters of long chain fatty acids that is derived from animal fats or vegetable oils” with an added requirement of having greenhouse gas emissions at least half of the baseline greenhouse gas emissions [4]. Biodiesel manufacturing attained extended attention and dramatic growth is observed in last decade as indicated in Fig. 1 [5, 6]. The characteristics like, lower GHG emissions, highly biodegradable molecular structure with minimal combustion toxicity, compatibility with existing engines and fuel distribution infrastructure are preferred features for its remarkable industrial growth [7, 8].

Generally, esterification/transesterification of free fatty acids/triglycerides with alcohol applying catalytic (chemical and biological catalysts) and non-catalytic are the principal reactions in prevailing biodiesel production [7, 9]. Among all the catalytic routes, biodiesel production using chemical catalyst is the most commercialized route due to shorter reaction time and high yield [10]. However, there are some limitations in chemical catalysis such as, catalyst recovery and recycling, excessive amount of alkaline wastewater and complexity of downstream product purification [11]. Additionally, the chemical catalytic process requires high-quality raw materials to save the process from saponification. Thus high-quality raw materials deliberately affect the process economics and increase the product cost [12]. Consequently, biocatalytic process has been recognized as a favourable alternative having mild reaction conditions, lesser wastes, easy purification and raw material flexibility [11]. Utilization of alternative low-cost raw materials such as second and third generation feedstock instead of using vegetable oils offers a potential way to reduce the biodiesel cost [13, 14]. Beyond biodiesel, research efforts have been also placed on new generation biofuel production from waste by integrating esterification reactions (enzymatic or chemical routes) with organic acids recovery from various waste resources. In addition, recent research has been also conducted to investigate the combustion performances of biodiesel blends in direct injection diesel engine, biodiesel derived from water hyacinth, palm biodiesel, *Garcinia gummi-gutta* biodiesel, tamarind biodiesel as well as alternative fuels blended with diesel [15–18].

A wide range of feedstock (edible, in-edible oil crops and waste oils, as well as microalgae), diverse reaction and separation conditions, and different types of catalyst make biodiesel manufacturing a complex system, which not only requires empirical work, but also the modelling research efforts. Recent comprehensive reviews by Muhammad et al. and Bhatia et al. and by Ananthi et al. provide very good overview of the research advancement in the biodiesel production including feedstock resources and characteristics, oil extraction and transesterification methods, reactor design and process intensification. To better manage and grasp the complexity of biodiesel manufacturing process, Process Systems Engineering (PSE) offers a solution by focusing on the development and application of the modelling and computational methods. This article shall henceforth a review on state-of-the-art PSE modelling research of biodiesel production and supply chains, and identify the emerging gaps and future research frontiers. Specifically, process design and simulation of different technologies for biodiesel production are compared in Sect. "Process design and simulation". Section "Sustainability evaluation of biodiesel production" focuses on the sustainability evaluation (economic and environmental aspects) of biodiesel production. Section "Optimization" discusses optimization of biodiesel production system at both processes and value chain design levels, which is followed by concluding remarks and critical perspectives for future research.

## Process system engineering in biodiesel production

**Fig. 2:**
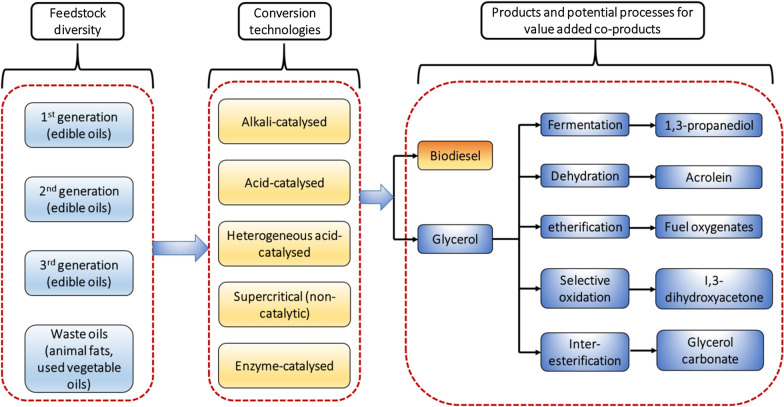
Schematic representation of technological choices and feedstock for biodiesel production

**Fig. 3:**
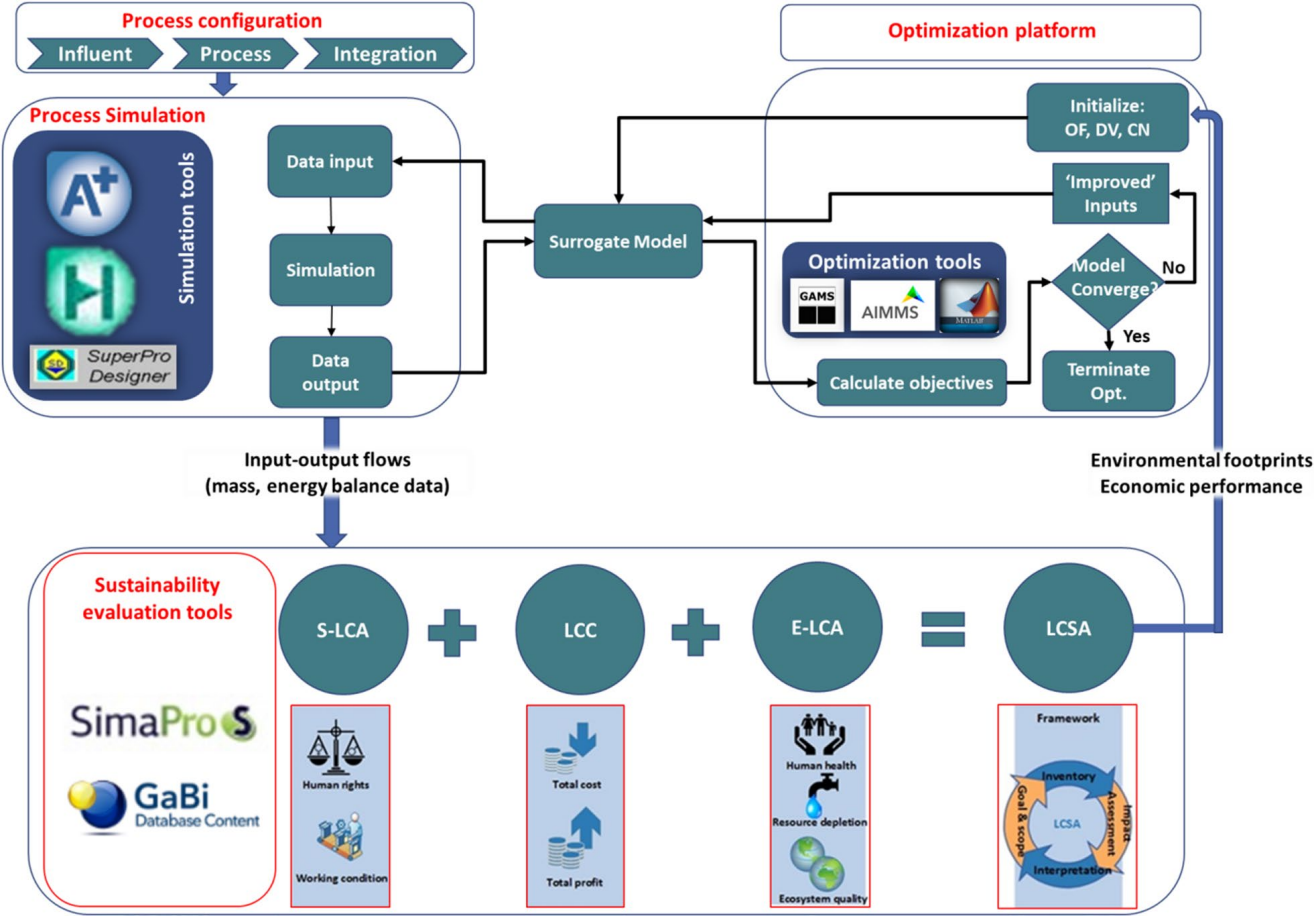
General framework for integration of different modelling tool

Despite the research advances and commercialization of quality biodiesel as drop-in biofuel in line with standard specification (EN 14214:2012 or ASTM 6751 12), biodiesel manufacturing still represents a complex system, which not only requires empirical work but also the modelling research efforts. A wide range of feedstock (edible, in-edible oil crops and waste oils), diverse reaction and separation conditions, and different types of catalyst have been used in biodiesel manufacturing (Fig. 2). Several modelling tools have been applied to tackle biodiesel manufacturing complexity including process design and simulation, sustainability evaluation and optimization (Fig. 3).

## Process design and simulation

Process simulation is a model-based illustration of physical, chemical, biological, and other unit operations and technical processes in a software. It can be used for the design, development, analysis, and optimization of biodiesel production processes.

The advantages of process simulation are to (a) reduce plant design time by allowing designers to quickly test various plant configurations; (b) improve current processes by answering ‘what if’ questions, determining optimal process conditions and assisting in locating the constraints in the process. The ultimate objectives of using process simulation are to realize faster troubleshooting, online performance monitoring and real-time optimization. A variety of modelling platforms, e.g. Aspen Plus, Aspen Hysys, SuperPro Designer, provide a resource where researchers and engineers can model, simulate, design their processes.

Several challenges arise for researchers when using these modelling platforms. The first challenge that researchers usually face is to define and select the appropriate chemical species taking part in the whole process. Yun et al. [19] added three different free fatty acids (oleic acid, stearic acid, palmitic acid) and one triglyceride (tri-olein) as model components to simulate the biodiesel production process from waste vegetable oil. A more comprehensive representation of the waste vegetable oil was compiled by Abdurakhman et al. [20] using five different triglycerides (tri-palmitin, tri-stearin, tri-olein, tri-linolein, tri-linolenin) and five FFA (linoleic acid, oleic acid, palmitic acid, stearic acid, linolenic acid) as model components. It was shown that the use of realistic feed compositions and sensitivity on the changes of composition is highly important to provide a more realistic assessment of the large-scale plant [20]. Due to the variable composition of biodiesel feedstock, the incorporation of all the components in a process simulator is also a challenging issue. Although several triglycerides with varying fatty acid chains are present in the Aspen Plus databanks but their physical property data are not well managed. Moreover, other components such as enzymes are still non-databank components. These components have usually undefined structures and/or difficult to characterize due to which their incorporation in process simulator is still a challenging issue.

The second challenge for using modelling platforms is to identify the available chemical and physical properties in the database. Modelling the biodiesel production system in these simulators, the NRTL or UNIQUAC thermodynamic models are usually selected due to polar compounds (glycerol, methanol and water) and the non-ideal nature of the transesterification reaction system. Zong et al. [21] applied chemical constituent fragment approach for the estimation of thermo-physical properties of triglycerides. This methodology was then extended to individual mono- and diglycerides [21]. In most of the simulation studies conducted for biodiesel production, UNIFAC method were employed which resulted in reliable prediction of the missing NRTL coefficients of trilolein–methanol and triolein–glycerol binary system [19, 22, 23].

Another challenge for using modelling platforms is to integrate solids, batch and custom processing unit modelling [24]. For example, biodiesel production involves several separation and FAME purification steps in which membrane is one option that can be utilized to obtain the desired product purity and recovery of recyclable materials (e.g. methanol, water, liquid lipase) [25]. Beside these challenges, several important data need to be gathered prior to process flowsheet design and simulation, e.g. reactor type and catalyst, rate of reaction or conversion, stoichiometry of reaction, process conditions, production capacity, mode of operation, etc. The approach that is often employed in the process design and simulation of production plants starts from reactor selection and proceeding outward by adding separation and recycle system [26, 27]. Among these several steps, the reaction procedure and the type of catalysts employed in the transesterification are crucial which determine purity of the product as well as severity of downstream separation and purification steps [28]. The following sub-sections present the process design and simulation of chemical and enzyme-catalysed biodiesel production processes along with the heat integration studies.

### Process design of biodiesel production using chemical catalyst

In the design of biodiesel production process, choice of operation mode for the process is one of the most important decision. Many publications on process design and simulation for biodiesel production are available (Table 1). These studies were based on the evaluation of heterogeneous and homogenous chemical catalysis as well as supercritical conditions (non-catalytic) in the context of process economy for a batch and continuous operations. Economic comparison of continuous and batch process for biodiesel production has been published by Sakai et al. [29]. Different types of catalyst (heterogeneous and homogenous alkali) and purification methods are compared extensively. Results elucidated that batch processes were more expensive than continuous process [22]. Comparing the behaviour, Fonseca et al. [30] showed that under the usual operating conditions, single continuous stirrer tank reactor (CSTR) is not capable to achieve the same productivity as batch reactor. However, arrangement of CSTRs in series is a viable pattern for mass production than batch process [30]. Despite the some advantages of batch processes, continuous process is the only choice for large-scale biodiesel production [23].

**Table 1:** Key features of reported simulation studies in biodiesel production

Feed definition (model compound)	Production process	Thermo-physical properties estimation and thermodynamic model	Reactor module	Plant capacity (tons/year)	Operation mode	Simulation tool	Refs.
Triolein	Homogenous and heterogeneous alkali-catalysed	–	Batch	7260	Batch	SuperPro Designer	[29]
Pure triolien + oleic acid	Alkali and acid-catalysed processes	NRTL and UNIQUAC-LLE	Yield	8000	Continuous	Aspen HYSYS	[31]
Pure triolien + oleic acid	Alkali, acid, heterogeneous acid-catalysed and supercritical processes	NRTL, UNIFAC-LLE and UNIFAC-VLE	Yield	8000	Continuous	Aspen HYSYS	[22]
Triolein and trilinolein	Supercritical process	NRTL and UNIFAC with Redlich–Kwong equation of state	Yield	8000	Continuous	Aspen PLUS	[43]
Triolein, tripalmitin and trilinolein	Supercritical with power cogeneration process	UNIFAC and Soave–Redlich–Kwong equations of state	–	2780 and 16,550	Continuous	CHEMCAD	[44]
Triolein	Enzyme-catalyed process	NRTL and UNIFAC-DMD	Stoichiometric	8000 and 200,000	Continuous	Aspen PLUS	[23]
Triolein, monoolein, stearic acid, palmitic acid, oleic acid	Enzyme-catalysed process	NRTL and UNIFAC	Stoichiometric	6482	Continuous	Aspen PLUS	[19]
Tripalmitin, tristearin, triolein, trilinolein and trilinolenin; palmitic, stearic, oleic, linoleic and linolenic acid	Alkali-catalysed process	NRTL	Conversion	64,000	Continuous	Aspen HYSYS	[20]
Triolein, diolein, monoolein	Enzymatic process	NRTL and UNIFAC-DMD	Kinetic (CSTR)	11,200	Continues	Aspen HYSYS	[45]

**Fig. 4:**
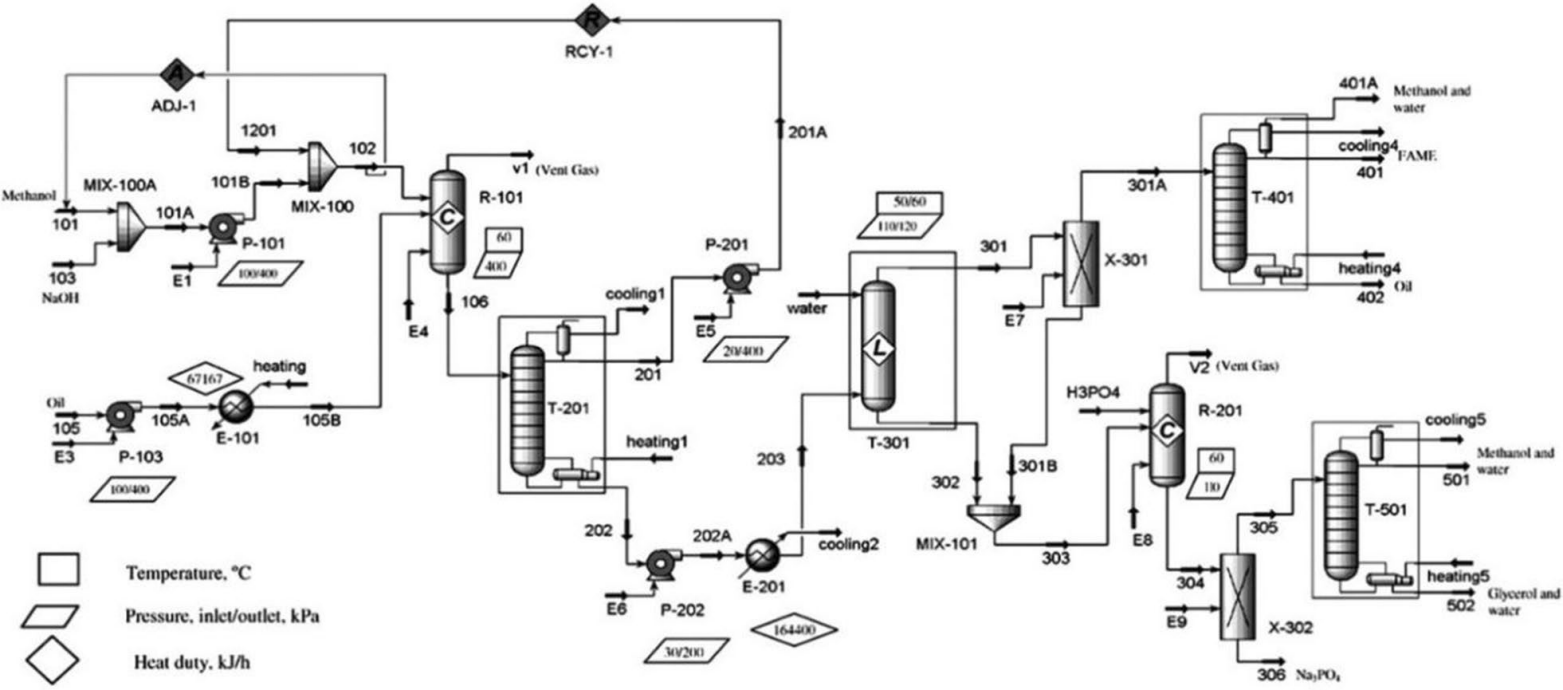
Flow diagram of alkali-catalytic route for biodiesel manufacturing using refined vegetable oil [31]

**Fig. 5:**
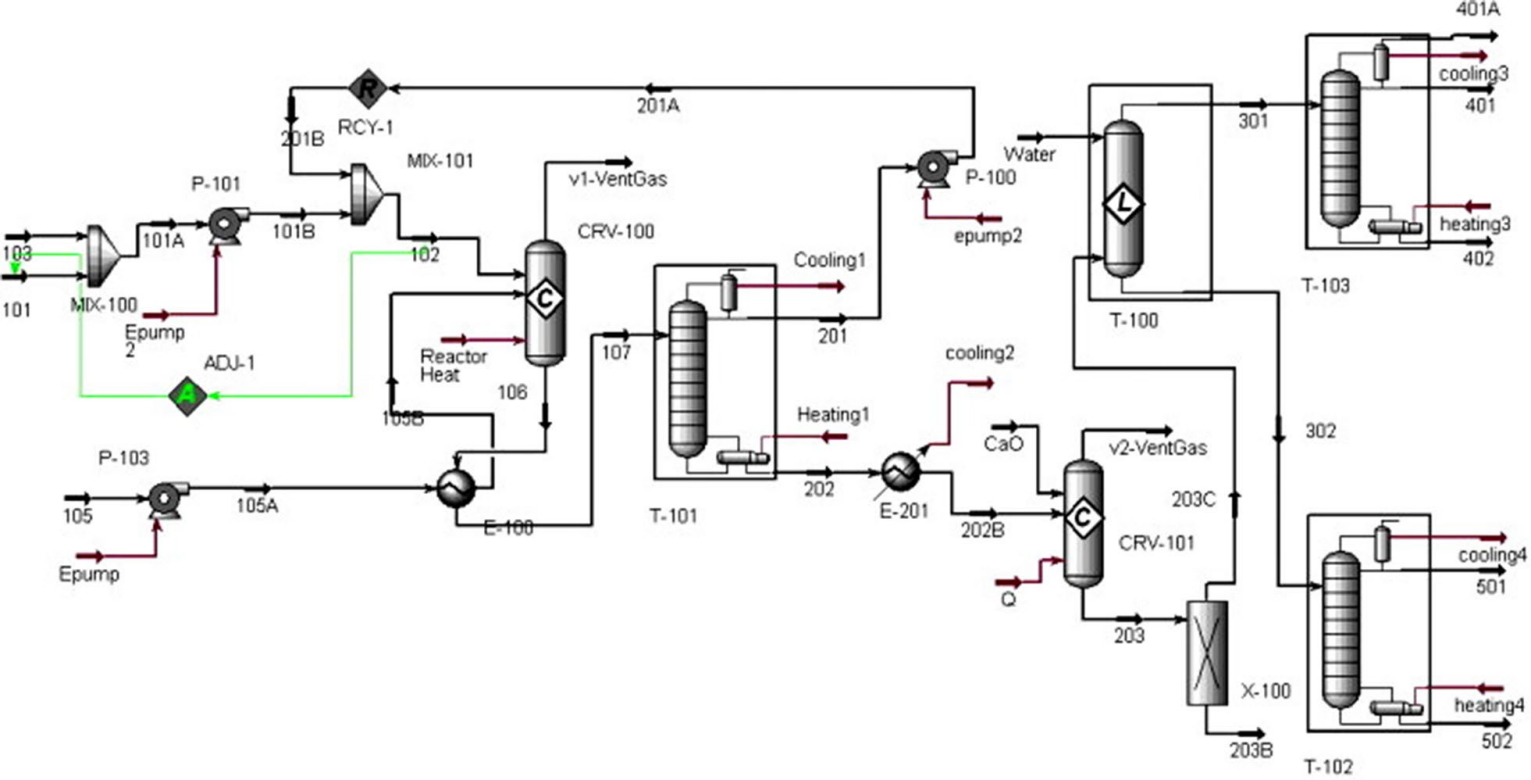
Flow diagram of homogenous acid-catalysed route for biodiesel manufacturing using waste cooking oil [22]

Regarding continuous production of biodiesel, Zhang et al. [31] attempted to design and simulate theoretical scale industrial plant using Aspen HYSYS. Various chemical catalysts (including homogenous-alkaline and acid catalyst) and feedstock (waste cooking oil and virgin vegetable oil) were used to investigate that how each type of catalyst and feedstock affect the process design. The unit operations included in the process design were transesterification, esterification, recovery of methanol, biodiesel separation and purification with either extraction of methyl esters using hexane or conventional water washing. The techno-economic feasibility of each technological option was evaluated and compared on the basis of material and energy consumption. The simulation results revealed that each process is distinct in their merits and demerits which are highly dependent on feedstock quality and the catalyst employed. Overall, alkali-catalytic process with virgin vegetable oil as a feedstock (Fig. 4) is a preferred option having less capital investment, but its operating cost is high because of high-quality feedstock requirement [31]. Modification in the design was carried out for low-quality oil (waste cooking oil) having high amount of FFAs. In this case, esterification of FFAs catalysed by sulfuric acid was carried out prior to the alkaline transesterification step. Contrarily to alkaline process with acid pre-treatment, the acid-catalysed process (see Fig. 5) was found suitable requiring no pre-treatment step. However, in this design, the larger methanol requirement resulted in larger reactor and distillation columns [18]. In addition, the presence of sulphuric acid requires a stainless steel reactor, which results in higher reactor cost.

**Fig. 6:**
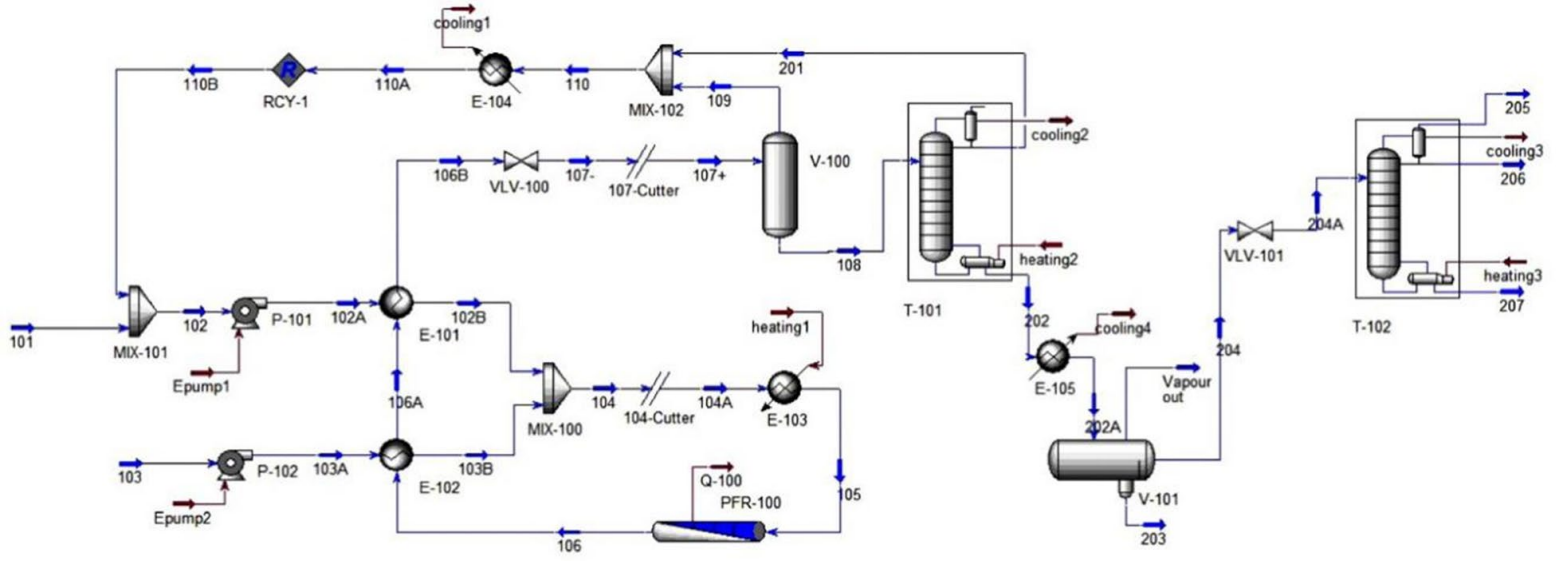
Flow diagram of non-catalytic (supercritical alcohol) biodiesel manufacturing route [32]

Heterogeneous acid-catalysed process and supercritical conditions (non-catalytic process) were also simulated in Aspen HYSYS by West et al. [18]. The simulation results were used to assess the performance of each process for low-quality feedstock. Results showed preference of non-catalytic (Fig. 6) and heterogeneous acid-catalysed process over alkali and homogenous acid-catalysed process due to reduced separation stages which results in lower capital investment. However, the process has high-energy profile due to heating and pumping.

From the above discussion, it is inferred that each process consists of the same process units (including reactors, washing column, distillation columns, heat exchangers and pumps), but the process operation for biodiesel production may differ due to type/purity of feedstock. Moreover, all the simulations studies proved that each process could yield high-quality biodiesel within definite process conditions. However, these simulation studies commonly lack integration of real industrial data, therefore, leading to under or overestimation of some of the simulated results for energy and mass balance. As an example, water consumption (11 kg/h of water is required to produce 1177 kg/h of biodiesel) and waste fractions estimated by Zhang et al. [31] are unrealistically low when compared to real industrial data (47.5 kg of water for 100 kg of biodiesel [23]). For any biodiesel process design and simulation, incorporation of actual industrial data is complementary to better analyse and reflect the process performance.

### Process design of biodiesel production using biocatalyst

**Fig. 7:**
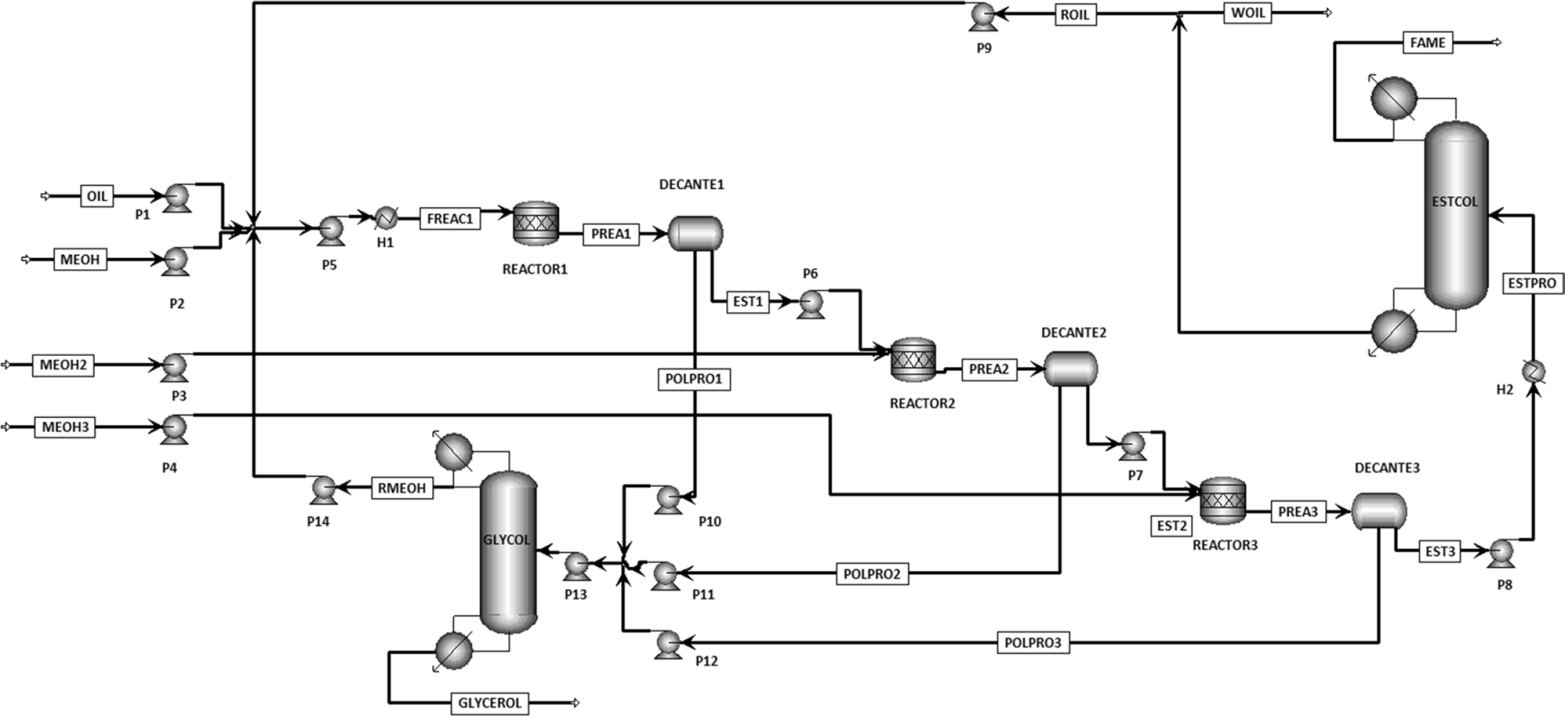
Flow diagram of solvent-free enzymatic route for biodiesel manufacturing [23]

**Fig. 8:**
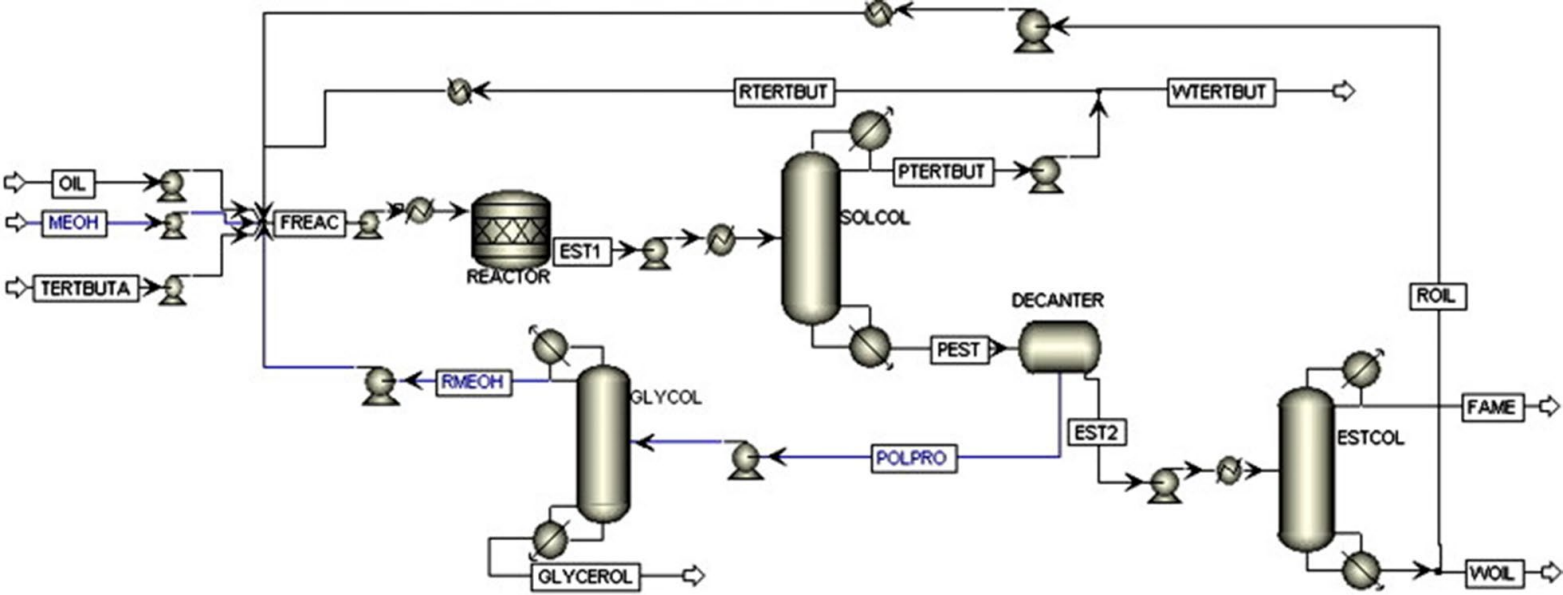
Flow diagram of co-solvent enzymatic route for biodiesel manufacturing [23]

Process design for industrial scale enzyme-catalysed biodiesel production is entirely different from the conventional setup. Enzymes are expensive and slow reacting species as compared to conventional chemical catalysts, but offer much simpler and easier purification scheme. Process design has been carried out by Harding et al. [33] and Al-Zuhair et al. [34] for enzymatic biodiesel, but the process lacks in optimization on some points. Sotoft et al. [23] extended the enzymatic process further by designing co-solvent and solvent-free operations for biodiesel (see Figs. 7, 8). Simulations was carried out in Aspen PLUS to explore how each operation affect enzyme performance and process design as well as the process economics. The solvent-free process was designed using three reactor modules in series with inter-stage separation of glycerol through decanters. This configuration made possible methanol stepwise addition, which is necessary to prevent enzyme deactivation by methanol. While in co-solvent process design, the required yield was achieved by employing only one reactor module. Distillation is used for methanol recovery and product purification in both processes. Solvent-free and co-solvent operations differ in solvent recovery requirements by distillation, which influence the process economy by making the process energy intensive. Zheng et al. [35] stated that the co-solvent process can be made energy efficient if distillation column is replaced with triple-effect evaporator for solvent and methanol recovery. Complete energy balance shows that enzyme-catalysed process is more energy efficient than alkali/acid and non-catalytic processes [35]. The co-solvent process was further enhanced by designing the enzymatic process with supercritical CO_2_ as a co-solvent [36]. Using supercritical CO_2_ was found more competitive eliminating the need for solvent recovery steps that are necessary in case of organic solvents. Contrarily to these models, Yun et al. [19] proposed two-step process design for enzymatic biodiesel production. They employed a wiped-film evaporator instead of distillation column to acquire the required purity of fatty acid methyl esters which must exceed 98.5%. A promising conversion efficiency was achieved by adding de-acidification step after transesterification. However, this adds additional cost incurring steps of neutralization and salt removal.

### Heat integration

In general, biodiesel production process requires a number of distillation steps for product purification (mainly with non-edible oil as the feedstocks) and to recover the methanol for recirculation. Pinch analysis [37] is the well-established method for heat integration in the process and optimal design of heat exchanger networks. Sanchez et al. [38] used pinch technique for heat integration in biodiesel manufacturing from microalgae. An optimal heat exchanger network was designed to reduce the load on external cooling and heating utilities. The simulation results showed that the heating and cooling utilities were reduced by ~ 13% and 11%. Meanwhile, Song et al. [39] reported that the operational cost of biodiesel from microalgae can be reduced by ~ 41.6% and 22.5% compared to two different reference cases when pinch analysis-based heat integration were performed. Yun et al. [19] put forward pinch analysis for heat exchanger network design and energy optimization of enzyme-catalysed biodiesel production process. The results showed a reduction in heating requirement by 15.6% compared to non-integrated process. Several other studies utilized pinch analysis for optimal heat and mass integration and found a significant reduction in energy consumption and utility cost [40, 41]. However, the thermodynamic approach adopted in these studies lack in configuration of subsystems which fails in guarantying best decisions [42]. In this regard, Martin et al. [42] made a contribution by the simultaneous heat integration and optimization approach for optimal process design of biodiesel. Apparently, the temperatures and flowrates were key decision parameters for both the optimization and the heat integration concern that resulted in much lower energy and water consumption with higher overall profit.

## Sustainability evaluation of biodiesel production

Sustainability, as alike concept to sustainable development, has been well thought-out to encompass the primary balance of three dimensions: environmental, economic and social, where poor performance related to one could impede performance on the others [46].

Life cycle sustainability assessment (LCSA) refers to the evaluation of all environmental, social and economic impacts in decision-making processes towards more sustainable products throughout their life cycles. Initiated from life cycle assessment, the life cycle thinking approach has been extended since 2002 to form a LCSA methodology framework, which consists of three pillars (Fig. 3)—environmental life cycle assessment (LCA), life cycle costing (LCC) and social-LCA. As a systematic and rigorous evaluation framework, life cycle sustainability provides integrative and holistic perspectives for multi-criteria decision on a given process or a system. As generalized in Eq. (1), LCSA accounts for all input–output flows occurring at each life cycle stage throughout the ‘cradle-to-grave’. Formalized by the International Organization for Standardization, LCA quantifies the environmental footprints associated with all stages of a product, service or process. LCC and SLCA examine the holistic economic aspects and social consequences respectively, evaluating the improvement opportunities of various product systems and processes including biodiesel:

1 $$E{I}_{\mathrm{kpi}}=\sum_{r}\sum_{s}{EIf}_{r,\mathrm{kpi}}^{\mathrm{in}}{X}_{r,s}^{\mathrm{in}}+\sum_{c}\sum_{s}{EIf}_{c,\mathrm{kpi}}^{\mathrm{out}}{X}_{c,s}^{\mathrm{out}},$$

where the variable $E{I}_{\mathrm{kpi}}$ denotes the total sustainability impacts of a given process expressed as key performance indicator kpi (e.g. global warming potential and economic costs). $E{I}_{\mathrm{kpi}}$ is determined by the characterization impact factors for input resource *r* ${(EIf}_{r,\mathrm{kpi}}^{\mathrm{in}}$) or emitted compound *c* (${EIf}_{c,\mathrm{kpi}}^{\mathrm{out}})$ and the input or output flows ${(X}_{r,s}^{\mathrm{in}} \mathrm{or} {X}_{c,s}^{\mathrm{out}})$ at life cycle stage *s*.

Evaluation of sustainability aspects have increasingly been reported for biodiesel production process during the last decade. However, most of the reports focused on the environmental and economic aspects of sustainability while omitting social aspect. The following sub-sections present a detail discussion on the techno-economic and environmental performance of biodiesel production processes.

### Economic evaluation

Economic performance is the most imperative factor for evaluating the sustainability of biodiesel production and plays a vital role in industrialization of any process. The higher production cost is the major challenge for biodiesel production scaling-up and its use as an alternate to petro-diesel [47]. However, an extensive research has been conducted during the past decades concerning the process economics and product cost reduction. These researches elaborated the utilization of different feedstock together with alternative technologies for the production and purification of biodiesel. Most of the studies analysed the total investment required for biodiesel production including fixed capital investment and production cost. Such cost estimation are often based on the process flowsheet and affected by the equipment type and size, construction material, material and energy balance [48]. Economic analysis can be performed in commercially available softwares such as Aspen In-Plant Cost Estimator or Aspen Icarus Process Evaluator [23, 32]. The key variables that determine the economic performance of a given biodiesel production plant include the production capacity, the type of feedstock, and the technological production process.

#### Production capacity

The production scale is the significant factor that could influence the techno-economic profiles by either decreasing or increasing the unit cost of biodiesel. This was elaborated by analysing the economic performance of biodiesel production plant with varying production capacities. One of such study was carried out by You et al. [49] for alkali-catalysed biodiesel production using refined soybean oil with three different production scales (8, 30, 100 kilo tons/year). It was concluded that higher capacity led to more attractive ARR (After-tax Rate of Return) with a lower BBP (Biodiesel Break-even Price) and higher NAP (Net Annual Profit). The author also stated that increase in plant capacity gave the same economic effects for soybean oil as well as waste cooking oil. On another hand, Apostolakou et al. [50] analysed the effect of plant capacity on the economic viability of biodiesel manufacturing using alkali-catalysed process. They found that production scale of 60 kilo tons/year is a threshold, above which, an increase in the production scale could improve the process viability since the production cost of biodiesel could be considerably reduced.

Similar result was reported by Van Kasteren and Nisworo [51] for supercritical process using used cooking oil with three different plant capacities (8, 80 and 125 kilo tons/year of biodiesel). They found that as the plant capacity increases, the biodiesel cost decreases from 0.52 to 0.17 US $/L. Glisic et al. [52] analysed the economics of the three different biodiesel production processes and investigated the effect of production scale on the net present value (NPV) of the process. The processes investigated were homogenous alkali-catalysed, non-catalytic transesterification for biodiesel production and catalytic hydrogenation process for diesel production. The authors reported that the plant capacity significantly affected the NPV of all processes. Especially for catalytic hydrogenation process, the NPV increased from 7 to 53.1 million US$ as the plant capacity were increased from 100 to 200 kilo tons/year. They concluded that plant capacity below 100 kilo tons/year (for all the investigated plants) results in negative NPV value after 10 years of project life.

Most recently, Navarro-Pineda et al. [53] assessed the economics of biodiesel production from jatropha using alkali-catalysed process. They also included the upstream process of jatropha plantation and pellet production from waste cake that is obtained from oil extraction process. The authors found that the biodiesel production cost remains constant when the production capacity was greater than 10,000 m^3^/year. However, at this scale, the plant expenses were greater than the plant income that can only be reversed by higher Jatropha oil yields. Similarly, Kookos [54] indicated that a plant with annual production capacity > 42,000 tons could produce economically competitive biodiesel utilizing spent coffee grounds as feedstock. As reported by Apostolakou et al. [51], the unit production cost of chemical-catalysed biodiesel decreases and can be expressed as the function of plant size. A significant decrease in production cost from 0.9 to 0.75 euros/L biodiesel was observed with the increase in production capacity from 0 to 40 kilo tons/year, which was followed by a plateau [50]. Contrarily, the total capital investment increases proportionally with production size but not linearly. Generally mass production is always cost-effective and most economical and same is the case with biodiesel. This effect of plant size on the total capital investment has been investigated in previous research [23] where similar trends were shown for co-solvent and solvent-free operation. It was observed that total capital investment varies between 10 and 60 million euros while plant size increases from zero to 1000 million kg biodiesel per year [23].

#### Feedstock

Most of the techno-economic studies concluded that the high cost of biodiesel production is mainly credited to the feedstock’s price. An economic assessment study published by Haas et al. [55] demonstrated that the biodiesel production cost increases linearly with increasing the cost of the feedstocks. They found that the cost of the feedstock is about 88% of the total biodiesel production cost. Thus, there was an increasing research attention on the low-cost feedstock as a measure to reduce biodiesel costs. However, the low-cost resource often represents low-quality feedstock, which incurs additional processing costs due to pre-treatment, separation and purification steps. For example, at industrial scale, the base-catalysed process is the most economically viable option to produce biodiesel from high-quality oils [32, 56]. However, it shifts to unfeasible solutions for low-quality oil feedstock (cheaper feedstock) containing high free fatty acids and water contents due to additional energy intensive pre-treatment requirement. A technology capable to process both low and high-quality oil feedstock without any additional pre-treatment steps offers a solution. Supercritical non-catalytic and enzymatic biodiesel production technologies are the examples of such technologies that have the ability to process low-quality feedstock without any pre-treatment requirements [19, 22, 23, 32].

#### Alternative technologies and their comparison for economical biodiesel production

The economics of biodiesel production vary with production technologies, which are driven by the number of unit operations and associated costs on equipment and energy consumption [47]. Alternatively, such economic advantages may also arise due to the relatively cheaper catalyst employed in the process. Moreover, catalyst type is highly important as it defines the type and sequence of production and purification scheme.

Table 2 compares the economic evaluation studies on different catalytic processes for biodiesel production. As previously mentioned, the alkali-catalysed process gives higher yields in shorter reaction time but it is not economically viable option when low-quality oil is considered [57]. It is limited by the saponification reaction (soup formation) that occurs between catalyst and free fatty acids, resulting in energy intensive downstream purification and making the process unprofitable. Acid-catalysed process avoids the side reactions and can esterify the FFAs to biodiesel. Zhang et al. [57] showed that acid-catalysed process could give lower production cost, lower biodiesel break-even price and better after-tax-return-rate compared to alkaline process using waste vegetable oil. However, the slow reaction rate, high alcohol requirement with larger reactor size and the corrosion problems imposed by the acid catalyst do have cost implications and makes the process economically unfeasible [22, 32, 57].

**Table 2:** Summary on economic parameters of different processes with different feedstock

Oil feedstock	Catalyst	Reaction media	Plant capacity (tons/year)	Operation mode	Production cost (million $/year)	Manufacturing cost (million $/year)	Biodiesel cost ($/kg)	Refs.
Chemical-catalysed processes								
Vegetable oil Waste cooking oil Waste cooking oil Waste cooking oil	Alkali Alkali Acid Acid (hexane extraction)	None	8000	Continuous	7.59 7.76 5.92 6.35	6.86 7.08 5.15 5.62	0.857 0.884 0.644 0.702	[57]
Waste cooking oil	Acid/alkali Homogenous acid Heterogeneous acid Non-catalytic	None	8000	Continuous	5.78 5.37 4.45 5.19	5.20 4.76 3.88 4.59	– – – –	[22]
Fresh canola oil Waste canola oil Waste canola oil	Alkali Acid/alkali Non-catalytic	None	40,000	Continuous	50.9 40.2 32.49	45.8 35 29	1.0 ($/L) 0.762 0.632	[32]
Rapeseed oil	Alkali	None	50,000	Continuous	65.9		1.15 ($/L)	[50]
Waste cooking oil	Non-catalytic	None	8000 80,000 125,000	Continuous		3.5389 17.134 18.790	0.52 ($/L) 0.24 0.17	[51]
Waste cooking oil	Non-catalytic Alkali	None	100,000	Continuous	54.934 55.590		0.727 ($/L) 0.671	[52]
Crude soybean oil	Alkali	None	37,854,118 L/year	Continuous	21.329	20.041	0.53 ($/L)	[55]
Palm oil	Alkali	None	1000	Batch	–	1166.7 ($/ton of biodiesel)	2.3 ($/L)	[59]

					Enzyme productivity (kg biodiesel/kg enzyme)	Enzyme price ($/kg)		
Enzyme-catalysed processes								
Rapeseed oil	Immobilized enzyme	None	8000 200,000	Continuous	1200	1000 10 1000 10	1.95 0.98 0.96 0.065	[23]
Palm oil	Immobilized enzyme	None	1000	Batch	5 cycles	1200	2.414	[59]
Waste cooking oil	Immobilized enzyme	None	8000	Continuous	200 cycles	–	1.15	[58]
Rapeseed oil	Immobilized enzyme	Co-solvent	8000 200,000	Continuous	4250	1000 10 1000 10	3.12 2.87 2.23 1.97	[23]
Waste oil	Immobilized enzyme	Co-solvent	8500	Continuous	–	2000 $/klU	0.86	[34]
Waste cooking sunflower oil	Immobilized enzyme	Supercritical CO_2_	8000	Continuous	–	800 €/kg 8 €/kg	1.64 €/L 0.75 €/L	[36]
Palm oil	Soluble lipase	None	1000	Batch	Single use	–	7.821	[59]
Vegetable oil	Soluble lipase	N/A	11,200	Continuous	Single use	1000	1.4	[45]

Heterogeneous acid-catalysed process could be a promising alternative with economic benefits compared to the homogenous acid-catalysed process. The techno-economic analysis performed by West et al. [22] showed that the heterogeneous acid-catalysed process has better economic performance (lower production costs and capital investment) compared to the homogenous acid-catalysed process which arises due to easy separation and recyclability of the catalyst, less corrosive nature and absence of washing steps for product purification. However, the slow reaction rate and lower biodiesel yields remain the major issues with acid-catalysed processes. These issues can be addressed by transesterifying the triglycerides with supercritical methanol. Using supercritical conditions give higher methyl ester yield in a shorter reaction time with reduced purification stages which results in very competitive biodiesel prices [32, 51] compared to previously denoted processes [22, 56]. The study carried out by Lee et al. [32] further elaborated the economic benefits of supercritical non-catalytic process by estimating the most promising values for discounted cash flow return rate (DCFRR), discounted payback period (DPP), and net present value (NPV) of the plant. However, the high alcohol requirement and extreme operating conditions (350 °C and 45 MPa) [22] makes the process energy intensive and incur considerable cost to the process.

Another perspective technology is enzyme-catalysed process that is more advantageous [23, 36] than chemical and non-catalytic processes in terms of milder reaction conditions, tolerating low-quality feedstock and easy purification of the products. The enzyme-catalysed process can also be carried out in the presence of solvent to increase the enzyme productivity. Sotoft et al. [23], demonstrated that the enzyme cost that was 50% of the raw materials cost in the absence of solvent was reduced to about 22% when *t*-butanol was used as a co-solvent. Although, the enzyme cost was significantly reduced but this led to the high production cost due to high energy consumption for solvent recovery. Using supercritical CO_2_ as a co-solvent can further improve the profitability of the process by both enhancing the enzyme productivity and eliminating the energy intensive step of solvent recovery [36]. This was confirmed by Lisboa et al. [36], reporting the production cost of biodiesel as 0.75 euro/L which is lower than the cost estimated by Sotoft et al. [23] (EUR 2.35/L of biodiesel) for solvent-free process with similar enzyme productivity and price. For low-quality oil feedstock, the enzymatic process is economically superior than the acid and alkali-catalysed processes in term of capital investment but inferior in operating cost [59]. This discrepancy was due to the high cost associated with the immobilized enzyme indicating that reusing the enzyme for several batches is needed to reduce the operating cost. Profitability of the process evaluated by net present value (NPV) for assumed interest rate of 13.5% and plant life span of 10 years showed that the enzymatic process is more economically attractive than the alkali-catalysed process [44]. Generally, the reusability of immobilized enzyme or using cheap biocatalyst (soluble or liquid lipase) are the most important aspects, improvements in which could make enzymatic process economically competitive with chemical-catalysed processes.

### Environmental evaluation

**Fig. 9:**
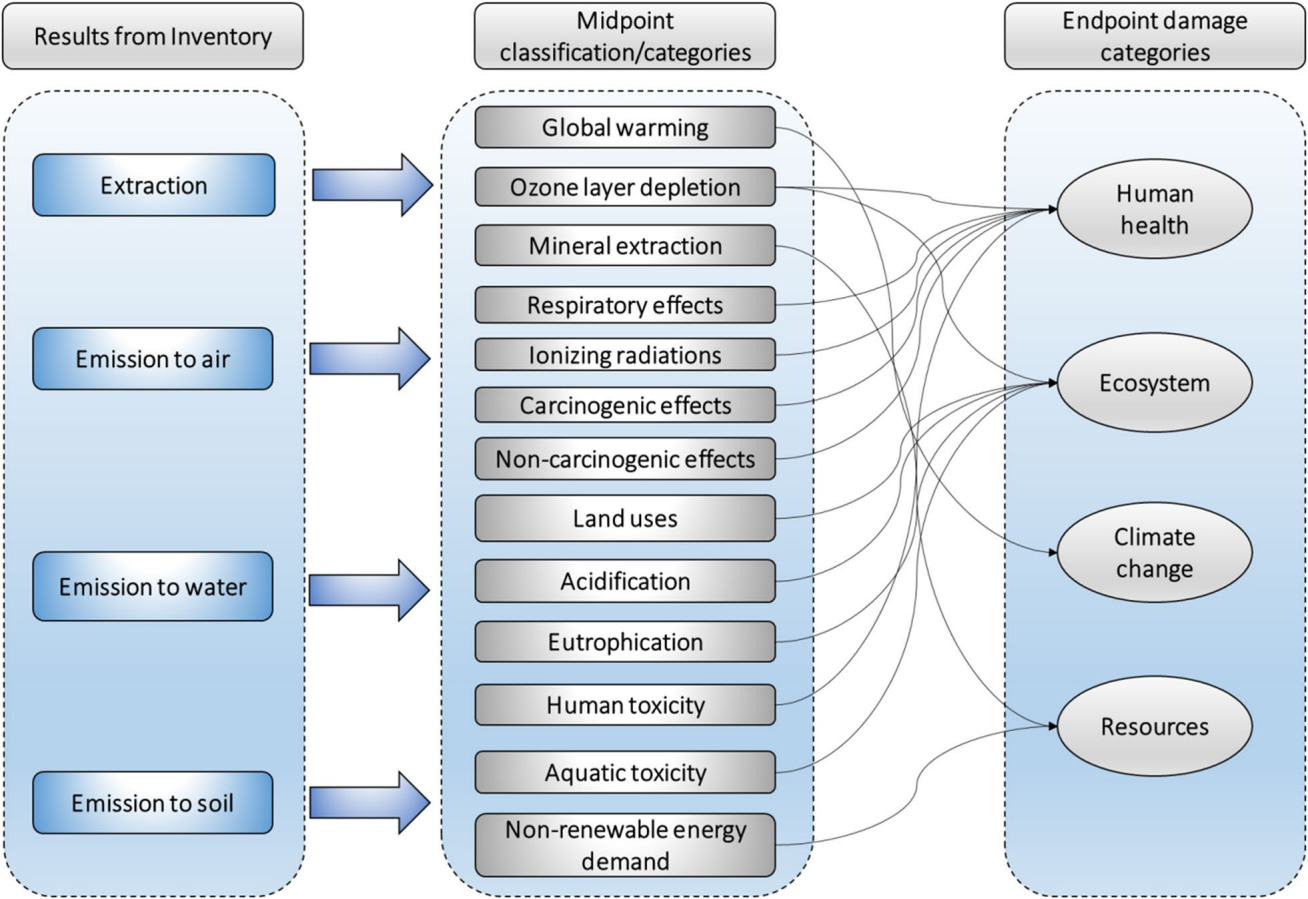
Life cycle impact assessment (LCIA) phase

Life Cycle Analysis (LCA) has been widely adopted as a tool to evaluate environmental performance of any product or process. In previous LCAs (see Table 3), the inventory of biodiesel production derived from computer-aided process were fed into LCA to identify environmental hot-spots contributing to the impacts and evaluate environmental sustainability of biodiesel production. As visualized in Fig. 9, the inventory including input–output flows are associated with mid-point environmental impact categories and converted to category indictors by using defined characterization factors; the aggregated indicator results provide characterized profiles of the biodiesel systems, which can be further normalized and linked with protection areas (i.e. end-point categories including human health, ecosystem, resource depletion).

**Table 3:** An overview of LCA studies focusing biodiesel production

Feedstock	Focus	Functional unit	Boundaries	Allocation	Impact categories	Refs.
Jatropha	Comparison of two technologies using differed catalyst	1 ton biodiesel	Well-to-wheels	–	Human health, ecosystem quality, resources	[76]
Rapeseed	Comparison of inorganic and biocatalytic production of biodiesel	1 ton biodiesel	Cradle-to-gate	Mass	ARD, GWP, FWAE, AP/EP, MAE, OLD, HT, TE, PO	[33]
Waste vegetable oil	Comparison of process alternatives	1 ton biodiesel	Industrial	Mass	ARD, AP, EP, GWP, MAE, OLD, HT, TE, PO, FWAE	[74]
Palm oil	Comparison of alkali and biocatalytic process	1, 5, 10 Mg biodiesel	Cradle-to-gate	–	CC, C, RO, RI, OLD, E, AP/EP, ME, R, LU, FF	[67]
Poultry fat Sewage sludge Beef tallow Waste cooking oil	Comparison of four production technologies from different FFA-rich wastes	1 ton biodiesel	Feed transportation and industrial	Mass	GWP, AP, EP, OLD, PO, NRED	[73]
Palm oil	Comparison of biodiesel technology using bio and alkali catalyst	1 ton biodiesel 1 ha palm oil	Agriculture and industrial	Mass	C, RO, RI, CC, R, OLD, E, AP/EP, LU, ME, FF	[70]
Waste vegetable oil	Biodiesel manufacturing	2018 kg biodiesel	Cradle to gate	–	GHG	[78]
Waste vegetable oil Soybean oil	Comparison of the environmental impacts from alkali and biocatalytic biodiesel production	1 ton biodiesel	Cradle to gate	Mass	ARD, GWP, OLD, TE, PO, HT, FWAE, AP, EP, MAE	[11]
Jatropha oil Waste vegetable oil	Comparison between jatropha oil and waste vegetable oil for biodiesel production using alkali-catalysed process	1 ton biodiesel	Cradle to gate	–	GWP, HT, RI, RO, OLD, TE, MAE, AP, EP, LU, NRED, ME	[65]
Soybean oil	Comparison between ethylic enzymatic and methylic alkaline routes for the production of biodiesel	1 ton biodiesel	Cradle to gate	–	NRED, GHG, OLD, PO, AP, LEG, SWG	[75]
Soybean oil Palm oil	Comparison of biodiesel production from palm and soybean oil	1 MJ biodiesel	Cradle to gate	Energy	ARD, GWP, HT, AP, EP	[66]
Soybean oil Jatropha Microalgae	Comparison of biodiesel derived from jatropha, soybean and microalgae	1 MJ biodiesel	Well to wheel	Mass energy	GWP, ARD, OLD, PO, AP, EP, HT, FWAE, MAE, TE	[60]

*ARD* abiotic resources depletion, *GWP* global warming potential, *MAE* marine aquatic ecotoxicity, *TE* terrestrial ecotoxicity, *OLD* ozone layer depletion, *PO* photochemical oxidation, *HT* human toxicity, *EP* eutrophication potential, *FWAE* fresh water aquatic ecotoxicity, *AP* acidification potential, *CC* climate change, *C* carcinogens, *RO* respiratory organics, *RI* respiratory inorganic, *E* ecotoxicity, *ME* minerals extraction, *LU* land use, *FF* fossil fuels, *NRED* non-renewable energy demand, *R* radiation, *GHG* greenhouse gas emissions, *SWG* solid waste generation, *LEG* liquid effluents generation

#### Key methodological challenges in LCA

Biodiesel production can be largely classified as three life cycle stages. Raw material production is the first stage, which includes cultivation, harvesting, transportation and storage of oil seed crops, as well as production and transportation of all the required chemicals. The second stage involves pre-treatment (milling, extraction and purification) of oil feedstock and conversion via esterification/transesterification to biodiesel. The third stage includes storage, distribution and transportation to petrol station, and eventual burning of biodiesel. As summarized in Table 3, LCA study conducted by Hou et al. [60] adopted a full well-to-wheel approach by including all relevant processes in the life cycle stages of biodiesel (e.g. production of chemicals and energy, feedstock cultivation and transportation, production of biodiesel and combustion of biodiesel at use phase). However, majority of the surveyed studies adopted well-to-gate approach (see Table 3) excluding the step of biodiesel distribution and end use. This approach is useful when the study is conducted to compare different technological pathways for biodiesel production, since the performance of vehicle engine does not change with the fuel combustion produced from different technological routes [61]. But, when the purpose of the assessment is to compare biodiesel with their fossil substitute, e.g. biodiesel with conventional diesel fuel, the well-to-wheel approach offers better reflection of the overall life-cycle performance where engine plays a role for exhaust gas emissions and ignition performance. Significant reductions in particulate matters, hydrocarbons and carbon monoxide emission are reported which are the profound advantages of biodiesel over conventional diesel [62].

Functional unit is another important factor which quantify the identified functions of a product system in which all the materials and energy flows and all effects resulting from these flows are related [63]. Mostly four types of functional unit can be identified in biodiesel LCA which include input-related units, output-related units, unit of agriculture land and year [64]. In biodiesel LCAs, majority of studies selected functional units based on the output of the product system (e.g. ton of biodiesel, L of biodiesel, MJ of biodiesel) [60, 65–67], while few studies used agricultural land and kilometres of transportation service as a functional unit [68, 69]. Besides, some studies presented the final results using multi-functional units [68, 70]. Ravindra et al. [70] used input, output and agricultural land related functional units. They used the product biodiesel as the output-related functional unit; for oil extraction functional unit is the production of 1000 kg of oil while functional unit for agriculture stage is per hectare of cropland. Similarly, Zhang et al. [69] reported two output-related functional units in their study for biodiesel based on the MJ of biodiesel and 1 km of driving distance. The implementation of kilometre of transportation service as a functional unit is better option when the goal is to compare biodiesel and fossil fuels used for transportation. Assessment with multiple functional units avoids biased outcomes and is highly effective for better assessment of any system in diverse scenarios.

Apart from functional unit and system boundary definition, the allocation approach, i.e. partitioning of environmental burdens among the multiple product is of great importance for biodiesel systems [63]. In biodiesel LCAs, the key allocation concern is between the biodiesel and by-product glycerol. There are mainly four options for adopting the allocation approach namely, null allocation, physical allocation, economic or market value allocation, and system expansion or substitution-based allocation [71]. Among the biodiesel LCAs surveyed in this review, the choice of allocation is dispersed (Table 3). The allocation adopted in most of the biodiesel LCAs were based on the physical properties of the product. Some studies related to biodiesel LCAs adopted the null-allocation approach and assigned all the environmental burdens to the main product biodiesel. However, this approach is not necessarily representative of the actual contribution of the studied products. Different allocation procedures may influence the results of biodiesel LCAs, which should be evaluated by sensitivity analyses [63]. Castanheira and Freire [72] analysed the sensitivity of the final LCA results to different allocation approaches in palm biodiesel evaluation. They adopted three different allocation methods (mass allocation, energy allocation and economic allocation) and stated that the environmental impacts estimated with energy and economic allocation were higher than those obtained with mass allocation. Our summary in Table 3 presents a lack of robustness analyses in the biodiesel LCAs, i.e. sensitivity analyses not presented in most of the published work.

A number of research articles have been published on the evaluation of environmental performance of biodiesel and its use by considering various feedstock and alternative production technologies. Following sub-sections discuss in detail the environmental performance of biodiesel utilizing various feedstock and different production technologies.

#### Environmental performance of biodiesel using various feedstock

A variety of feedstock can be utilized for biodiesel production that offers environmental benefits based on their requirements for agriculture, transportation and several other conditions. The feedstock assessed for biodiesel environmental performance through its life cycle includes first, second and third generation feedstock along with waste oils and fats (see Table 3). Hou et al. [60] conducted a comprehensive LCA of biodiesel from different feedstock (soybean, jatropha, microalgae) and compared the environmental performance with conventional diesel (fossil-derived). Among different feedstock, microalgae come out as more feasible alternative in terms of terrestrial eco-toxicity potential (TEP) and fresh water aquatic ecotoxicity potential (FWAEP) due to lower agriculture inputs. Hou et al. [60] found that FWAEP that is caused by agricultural process contributed 92%, 43.9% and 91% to the total environmental burden in the life cycle of jatropha, microalgae, and soybean-based biodiesel, respectively. In comparison to conventional diesel, biodiesel performed better in terms of global warming potential (GWP), ozone layer depletion (ODP) and abiotic depletion (ADP), but showed worse performance in acidification, eutrophication, photochemical oxidation, and toxicity [60]. The better performance of biodiesel in ADP, GWP and ODP is principally due to CO_2_ uptake and solar energy from the environment through photosynthesis during the biomass agriculture. In another study, the environmental performance of second-generation biodiesel was compared with waste oil-based biodiesel [65]. When non-edible oil from jatropha is compared with waste cooking oil for biodiesel production, the latter showed lower environmental impact to all damage categories (climate change, human health and ecosystem quality). The inferiority of jatropha-based biodiesel in environmental performance is attributed to fertilizers, chemicals, water and land requirements for biomass cultivation and harvesting [65]. However, waste cooking oil-based biodiesel showed severe environmental impact for damage categories of resources (including mineral extraction and non-renewable energy demand). The total burden on the environment was 74% lower in case of utilizing waste vegetable oil as a feedstock compared to jatropha oil [65].

Further to compare environmental impact of a variety of waste feedstock, Dufour et al. [73] adopted well-to-gate analysis of feedstocks including beef tallow, sewage sludge, poultry fat and waste vegetable oil. The scope of the study was further extended by conducting well-to-wheel analysis of first-generation feedstock (soybean and rapeseed) to compare the impacts of waste oil derived biodiesel with first generation and conventional diesel. When these findings were compared, results elucidated the environmental superiority of FFA-rich materials derived biodiesel compared to both first-generation biodiesel and conventional diesel. While, among FFA-rich feedstock, waste vegetable oil showed better environmental performance in terms of GHG savings [73]. It can be conferred from the above discussion that waste oils are paramount encouraging feedstock for biodiesel production.

#### Environmental performance of biodiesel from chemically catalysed technological routes

Besides comparing different potential feedstock, LCAs were also conducted on the perspective of comparing different technological pathways for biodiesel production. One of such study was conducted by Morais et al. [74] to evaluate environmental viability of biodiesel produced from three technological alternatives including non-catalytic process (supercritical) with propane as a co-solvent, acid-catalysed process, and traditional alkali-catalysed process with acid pre-treatment. For each of the alternative technology, depletion of abiotic resources and marine aquatic ecotoxicity potential were found the most relevant environmental impact categories. Methanol that is used as a raw material in all alternative processes, significantly contributed to the depletion of abiotic resources since it is synthesized from fossil resources. Compared to methanol, ethanol could be a preferred option due to its renewable origin. That is, ethanol is responsible for absorbing significant amount of CO_2_, decreasing significantly the GHG effect of the manufacturing system [75]. Beside this, non-catalytic (supercritical conditions) route using propane as a co-solvent is relatively more environmentally favourable process [74]. This is because of the absence of catalyst and its lower steam consumption compared to other process.

While, the acid-catalysed route generally causes the highest environmental impact, mainly due to high energy profile related with methanol recovery operation. Compared to alkali-catalysed process, the supercritical non-catalytic process was reported to reduce the acidification by 754%, abiotic resource reduction by 313%, marine aquatic ecotoxicity by 793%, and global warming by 496% [74]. When the environmental impact of alkali catalyst (potassium hydroxide and sodium hydroxide) is compared, sodium hydroxide (NaOH) exhibited greater environmental impact on ecosystem quality and human health [76]. This can be explained by the sodium hydroxide that is an environmental hazardous material as compared to potassium hydroxide (KOH). Moreover, NaOH produces water-soluble salts on neutralization with acid and KOH precipitated to potassium sulphate by reacting with sulphuric acid. Salt precipitation decrease the overall water consumption and discharge of polluted water to environment, while this is not the case in using NaOH [23].

#### Environmental performance of biodiesel from enzyme-catalysed technological routes

In contrast to aforementioned studies, many researchers evaluated enzymatic technology for biodiesel production in their LCAs and reported that this technology has potentially lower environmental impact as compared to chemical catalytic technologies. For example, using biocatalyst (phospholipase) for degumming vegetable oils could reduce 44 tonnes of equivalent CO_2_ per 1000 tonnes of oil produced because of high efficiency and low raw material requirement [77]. To further elaborate the environmental benefits offered by enzymatic production of biodiesel, LCAs were conducted to compare enzymatic process with alkali-catalysed process. These studies showed that enzyme-catalysed process outperforms the alkali-catalysed process in each measure of potential impact categories including human toxicity, global warming, and depletion of ozone layer [33, 70]. Ravindra et al. [70] compared the results for both processes based on the single score and final total score. The single score result pointed out that, for both processes, the land use contributes the most to the environmental impact (75% for enzyme-catalysed and 70% for alkali-catalysed). However, the total score indicated less contribution to the total environmental impact by the enzyme-catalysed process [70]. Using immobilized enzyme instead of free enzyme in biodiesel production was found to further reduce the environmental burden on the processes [67]. This is because the reuse of immobilize lipase reduces consumption of minerals and carbohydrates needed for its soluble form production.

**Fig. 10:**
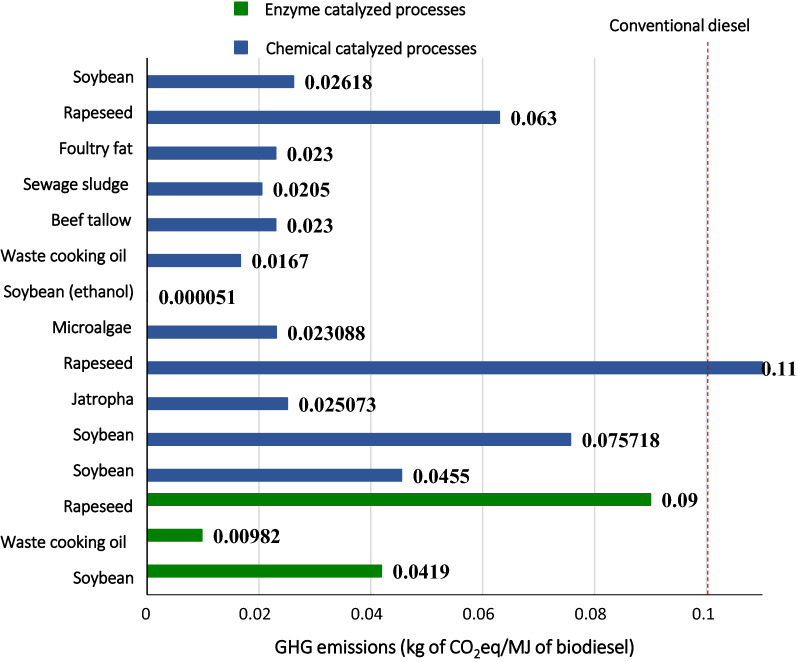
GHG emissions in surveyed biodiesel life cycle studies [11, 29, 60, 67, 73, 75] (conventional diesel [68])

Overall, the enzymatic production technology provides significant reduction in environmental impacts compared to chemical-catalysed processes. However, photochemical ozone creation, global warming potential, terrestrial ecotoxicity and human toxicity potential are some of the impact categories in which enzymatic process shows almost same contribution as the conventional alkali-catalysed process [11]. These impact categories can be made lower for enzymatic process when the agriculture stage is avoided and a low-cost waste vegetable oil is used as a feedstock. In a study, it was estimated that for one tonne biodiesel production, 1775, 1633 and 383 kg of CO_2_eq is emitted to the atmosphere by alkali-catalysed, enzyme-catalysed, and enzyme-catalysed using waste cooking oil, respectively [11]. The latter process shows significant reduction in greenhouse gas emissions. Figure 10 shows greenhouse gas emissions for biodiesel in the surveyed LCA studies in this review (see Table 3). Generally, GHG emissions range from 0.51 × 10^–4^ to 0.11 kg CO_2_eq/MJ of biodiesel, which is in most cases lower than the conventional diesel ensuring net GHG reductions for using biodiesel as a substitute to petro-diesel. The variation in GHG emissions with the same technology and utilizing the same feedstock can be attributed to the variation in the system boundaries, allocation methods and other methodological assumptions. For most of the cases, enzymatic processes show considerable reduction in GHG emissions compared to chemical-catalysed processes, which is probably due to the decrease in energy consumption. Comprehensively, it is inferred that the enzymatic process is more environmental benign process as compared to the chemical-catalysed processes.

## Optimization

The biodiesel production is an inherently complex system involving diverse feedstock, a number of technological alternatives, and various separation/purification sequences and conditions that require optimization on several aspects. In such complexity, conflicting design criteria can be concerned such as cost effectiveness and environmental sustainability. This section focuses on the optimization of biodiesel production considering the system complexity and sustainable design criteria, e.g. profit maximization, cost minimization, and environmental impact minimization.

### Single-objective and multi-objective optimization

Optimization approach has been applied to biodiesel production system at both processes and value chain design levels, which provides solutions and insights into decision-support. In previous research, as summarized in Table 4, a range of tools (Aspen Plus/HYSYS, SuperPro Designer, MATLAB, Excel) has been adopted to optimize biodiesel production process for multiple objectives. The methodology in these works is based on the implementation of the process model in the commercial process simulators (e.g. Aspen PLUS/HYSYS, SuperPro Designer) that are coupled with the multi-objective optimization (MOO) algorithm solving the process model for multiple objectives. Patle et al. [79] used Non-Dominated Sorted Genetic Algorithm (NSGA) executed in Excel. The algorithm was linked to rigorous process simulation in Aspen Plus for MOO of the two different continuous biodiesel manufacturing processes. The link and communication between Excel-based MOO programme and Aspen Plus was established via visual basic application (VBA). The optimization problem was solved for multiple objectives including profit, heat duty and organic wastes. The obtained results enabled them to decide the best production technology for a specific weighting of objectives.

**Table 4:** Multi-objective optimization in biodiesel production

Feedstock	Catalyst	Objectives	Optimization method	Simulation tool	Optimization tool	Refs.
Sunflower oil	Alkali	Product purity and energy consumption	MOGA	Aspen PLUS	modeFRONTIER	[83]
Waste canola oil	Acid	Profit and waste	Multi-objective Simulated Annealing Algorithm (MOSA)	Apen HYSYS and SustainPro	ENVOPExpert	[84]
Vegetable oil	Alkali	Profit, product purity, yield and energy consumption	NSGA	–	Matlab	[85]
Waste cooking oil	Alkali, acid	Profit, energy consumption and organic wastes	NSGA	Aspen PLUS	Excel worksheet with Visual Basic Application (VBA)	[79]
Soybean oil	Alkali	Economic, environmental, social	MOGA	SuperPro Designer	Matlab	[80]
Waste cooking oil	Alkali	Profit, fixed capital investment and organic wastes	MODE-TL	Aspen HYSYS	Excel worksheet with Visual Basic Application (VBA)	[82]

Similarly, Woinaroschy et al. [80] presented the multi-objective optimization of biodiesel production process considering three objectives (profit, volatile organic emissions, and number of jobs) for optimization. They used multi-objective genetic algorithm (MOGA) implemented in MATLAB that are linked with rigorous process simulation in SuperPro Designer. The Component Object Module (COM) feature of SuperPro Designer and MATLAB Graphical User Interface (GUI) were used to establish the link between SuperPro Designer and MATLAB for the data transfer. In this work, all the three pillars of sustainability (Environmental, economic, and social) were evaluated and optimized simultaneously. The evolutionary algorithms applied in these works perform well and have attractive properties when integrated with the process simulator. However, regarding the complexity of biodiesel production process, careful analysis of numerous problems is needed, including the constraints integration; entire flowsheet initialization; decision variables and their boundaries selection. Moreover, these algorithms face difficulty in applications where the model flowsheet demands a long time for convergence or fails to converge due to some values of decision variables suggested by the optimization algorithm as iterations proceeds [81]. Sharma et al. [82] adopted an attractive alternative to overwhelm these curbs and further reduced the computational means used during optimization. They used multi-objective differential evaluation algorithm with taboo list (MODE-TL) for the optimization of biodiesel production process from used vegetable oil [82]. Generally, the literature summarized in Table 4, adopted sequential modular simulation approach relying highly on the detailed equations for unit operations, which impedes the smooth application of derivative-based deterministic optimization solvers. Therefore, derivative-free optimization algorithms are mostly attractive due to their performance efficiency in discontinuous, non-differentiable, or highly non-linear expressions.

### Biodiesel process synthesis and optimization

Mathematical programming has been widely applied to optimize biodiesel process synthesis problem, considering the economic trade-offs and interactions among subsystems. By far the most systematically considered synthesis problems in biodiesel production is the heat exchanger networks synthesis, separation sequences, and superstructure optimization for alternative technologies [41, 86, 87]. This methodology was implemented by Martin et al. [42] to perform superstructure optimization of biodiesel manufacturing from microalgae and waste vegetable oil. A mixed integer non-linear programme (MINLP) is formulated and solved for five different technologies including enzymatic catalysis, alkali catalysis, acid catalysis, heterogeneous basic-catalysed, and under non-catalytic conditions (supercritical). The superstructure optimization was performed to find out the best option among different alternatives. The results indicated that when waste vegetable oil is utilized, the enzymatic technology is the best option yielding biodiesel with a production cost of approximately 0.6 US$/gallon, energy and water consumption of 1.9 MJ/gallon and 0.3 gallon/gallon of biodiesel, respectively. Similarly, for microalgae the best production process was the alkali-catalysed with a production price of 0.4 US$/gallon of biodiesel requiring 0.60 gallon/gallon of water and 1.94 MJ/gallon of energy. Similar to Martin et al. [42], Mansouri et al. [88] also used superstructure optimization but they included process intensification options in their framework to model biodiesel production from pure and waste palm oil. A more comprehensive superstructure optimization for process synthesis of microalgae based biodiesel was performed by Rizwan et al. [87]. They included all the possible alternatives for microalgae harvesting, pre-treatment and lipid extraction along with the possible alternatives for the transesterification technologies in their superstructure mapping.

### System and value chain optimization

Supply chain management (SCM) is relatively new optimization area that targets to integrate production plants with their suppliers and customers in an effective manner [89]. For biodiesel production, optimal design, management and integration of supply’s operations, manufacturing as well as distribution activities (entire supply chain) are crucial, to hasten transition towards large-scale and economically sustainable biodiesel [90]. Generally, biodiesel supply chains are multi-echelon networks including feedstock facilities, feedstock collection and pre-processing facilities, biodiesel production facilities, biodiesel distribution centres, and biodiesel consumers [91]. In addition, logistic framework is managed to facilitate efficient and substantial material flow between different echelons within the network. The most critical and important decisions for biodiesel supply chain network design are the location and optimum number of facilities, volumes of facilities, technological options, suitable logistics and carriage means, and optimum material flow.

Table 5 summarizes the state-of-the-art literature based on the type of feedstock used, decision variables addressed, uncertainty consideration, optimization approach used. Developing the first-generation biodiesel intensify the food crises. Therefore, design for second-generation biodiesel supply chains from waste oils and non-edible energy crops was focused [89]. Moreover, researchers carried out supply chain design for hybrid first, second and third generation biodiesel with consideration of land competition between edible and in-edible energy crops [92, 93]. An optimal design of biodiesel supply chain using multi-period mixed integer linear programming (MILP) model was developed in Argentina that considers land competition among different feedstock including soybean, sunflower and jatropha [92]. The results indicated that jatropha serves as a more promising feedstock alternative to edible or more valuable feedstock for biodiesel production.

**Table 5:** An overview of biodiesel supply chain studies

Feedstock	Decision variable	Uncertainty	Objective function	Optimization approach	Year-region	Refs.
Corn	Allocation decisions Capacity of facility Location of facility	–	Minimize total cost	MILP	2011-Brazil	[95]
Microalgae	Allocation decisions Capacity of facilities Technology selection	–	Minimize total cost	MILP	2015-South Korea	[96]
Sawmill waste Agricultural residues Forest residues	Allocation decisions Selection of technology Facility location Technology selection Allocation decisions Amount of production	–	Maximize net present value	MILP	2016-Germany	[97]
Soybean Sunflower Jatropha	Capacity of facilities Location of facilities Allocation decisions Technology selection Amount of production Transportation mode	–	Maximize net present value	MILP	2012-Argentina	[92]
Soybean	Selection of technology Facility location Allocation decisions Capacity of facility Inventory holding	Biodiesel demand Biomass availability	Total profit maximization	MILP	2016-	[102]
Microalgae	Location of facilities Capacity of facilities Allocation decisions	Resources supply Biodiesel demand Technical factors Cost parameters	Minimize total costs	MILP	2016-Iran	[100]
Jatropha, used cooking oil	Transportation mode Production capacity Facility location Capacity of facilities Inventory holding Allocation decisions	Environmental and cost parameters	Minimize environmental impact Minimize total costs	MINLP MODM	2017-Iran	[93]

Biodiesel supply chain has been developed considering different aspects of strategic level such as technology selection, location of facility and capacity determination [94]. Mixed integer programming (MIP) is mostly applied to solve biodiesel supply chain design and optimization problems. Considering strategic level decision-making, Leao et al. [95] formulated a MILP mathematical model to design biodiesel supply chain networks in Brazil. The model considered agricultural, logistics, social as well as industrial aspects for biodiesel manufacturing from castor oil. Supply chain networks for biodiesel were also designed for 2nd and 3rd generation feedstocks on the strategic level [96, 97]. Hombach et al. [97] exploited 2nd generation feedstock such as sawmill wastes, agricultural residues, and forest residues in their supply chain model incorporated with European biofuel regulations. To prevent sub-optimal solutions, tactical level decisions (like inventory level and production capacity in different periods) can be incorporated with the strategic level decisions. In this regard, Babazadeh et al. [93] designed biodiesel supply chain network by integrating both tactic and strategic level decisions in the supply chain model. Apart from minimizing the environmental burden of all the processes involved, the proposed model was effective only in minimizing the cost of biodiesel supply chain from feedstocks supply centres to consumers. As a result, high investment cost is obligatory to reduce the environmental burden. Although the integrated model prevented sub-optimal solution, it increased the level of complexity and in consequence needed more computational efforts than the non-integrated one [93].

The aforementioned studies mostly presented deterministic models by assuming known parameters in the supply chain network model. However, uncertainty is an intrinsic portion of every genuine system and can seriously pose the decision-making process. Overall, uncertainty of biodiesel supply chains can be classified as process uncertainty, demand and supply uncertainty [90]. Dal-Mass et al. [98] considered price uncertainty in designing biomass supply problem by describing distinct scenarios for price variations. In variance to Dal-Mas et al. [98], Kim et al. [99] considered all the three categories of uncertainty in biodiesel supply chain optimization. Shayan et al. [100] presented a two-stage robust MILP model under variant uncertainty sets. The model considered biodiesel demand, cost parameters, uncertainty in resource supply. When the decision-maker needs to cope with uncertainty but without sufficient historical data, the robust programming approach could be applied. In this context, Babazadeh et al. [101] presented a possibilistic programming approach to design a biodiesel supply chain network sourcing from waste cooking oil and jatropha. They addressed both cost and environmental uncertainties in a novel possibilistic programming, structured as MINLP model.

The above discussion suggests that supply chain optimization has been studied systematically at both tactical and strategic levels. Moreover, cost criteria is the most considered objective function considered so far (Table 5). Conversely, the social and environmental apprehensions are often overlooked. Moreover, research challenge in addressing the uncertainties in biodiesel supply chain design remains open. Through a thorough literature review on wider biofuel and bioproduct systems beyond biodiesel, a range of promising supply chain optimization research has emerged which deserves future research attention in biodiesel system optimization:

- demand-driven supply chain integration, in particular biodiesel with value-added platform chemicals derived from the same oil feedstock;
- supply-driven supply chain integration for multiple oil feedstock streams with similar processability, e.g. terrestrial oil crops integration with algae;
- waste value chain design under uncertainty considering the high variance in waste oil stream composition and supply;
- sustainable value chain optimization for biodiesel systems considering conflicting sustainability design criteria applying life cycle approaches.

## Conclusion/future prospects

Recent developments in biodiesel production suggest that the production of biodiesel offers evident environmental benefits but its economic competitiveness highly depend on feedstock sources, technological choice and production capacity. Further research is necessary in modelling areas to enable a sustainable biodiesel production. Our literature review also highlights several frontiers for future research and developments.

Due to the dominant role of feedstock in cost profiling, the selection of the low-cost feedstock is of importance for the development of economically feasible yet sustainable biodiesel production process. A life cycle approach, which addresses economic and environmental aspects, offers a holistic evaluation to highlight the improvement spaces and screen the suitable feedstock and technology options. Moreover, LCSA accounts for three sustainability aspects and provides systematic insights into decision spaces; LCSA could enable further investigation and decision to be effectively focused on the major hot-spots that can be further optimized to achieve sustainability trade-offs.

The biodiesel production process requires in-depth investigation to tackle multi-scale multi-criteria design challenge. Our literature review suggests that supply chain and process network optimization are generally based on discretized time intervals, which consider process design scenarios. Such approach represents a trade-off between solution quality and computational complexity. Surrogate-based optimization could reduce the computational complexity. Specifically, surrogate modelling techniques could be applied which follow a black-box or grey box approach and use first-principle modes as a source of computational experiments; the generated sample data points can be fit into surrogate functions to represent the accuracy of first-principle modelling and project process performances. This will enable the inclusion of technology alternatives (surrogate models) and resources for biodiesel production in a multi-objective optimization framework, considering decision variables and sustainability criteria at both process and network levels.

Despite the supply chain optimization research, much attention has been placed on the long-term planning. Mid- or short-term production scheduling problems emerged as a research gap in response to recent digital technology and data advances (Internet of Things, Smart of Machinery, Big Data). Such advances enable real-time data collection and have the potential to catalyse transformation of biodiesel refinery towards batch manufacturing modes. Thereby, batch scheduling to enable ‘production-on-demand’ biodiesel refinery represents an interesting direction.

Deterministic optimization has been the research focus, whereas biodiesel optimization under uncertainty emerged as an interesting research direction. Particularly, biodiesel is sourced from natural sustainable resources and relies on policy intervention (e.g. green technology deployment policy); thus, its production is regulated by seasonal variables and other uncertain factors. Under this context, the uncertainty performances of biodiesel production at single sites and multi-sites would be of particular interests. The potential uncertainty indicators include responsiveness and resilience. Responsiveness considers the biodiesel production performances in response to operational uncertainties (e.g. feedstock supply and diesel demand fluctuation); whereas the resilience indicates the system capacity to recover, adapt facing the unexpected external disruption (e.g. natural extreme events or policy intervention). Responsiveness and resilience in biodiesel production design has not yet been explored. By integrating the risk mitigation and resilience-building measures into the stochastic and/or robust optimization, precision decision-making presents a future optimization direction for biodiesel research.

## Data Availability

The data supporting the results of the article are included in this manuscript.
